# Tongnao Decoction (TND) Alleviated Atherosclerosis by Playing Lowering Lipid, Anti-Inflammatory, and Antioxidant Roles

**DOI:** 10.1155/2022/6061197

**Published:** 2022-05-25

**Authors:** Hui Jiang, Jianli Wang, Li Sheng, Xiaoyu Xu, Bingmei Zhou, Luying Shen, Minghua Wu

**Affiliations:** Department of Neurology, Affiliated Hospital of Nanjing University of Chinese Medicine, Jiangsu Province Hospital of Chinese Medicine, Nanjing 210029, China

## Abstract

**Background:**

Tongnao decoction (TND) has been extensively prescribed for the treatment of stroke. However, little is known about the role of TND in the progression of carotid atherosclerosis.

**Methods:**

A mouse carotid atherosclerosis model was established with a silastic collar placed around the right common carotid artery and fed on Western diet for 12 weeks. The treatment group was given a gavage of TND at a dose of 1 mg/kg/d. The atherosclerotic lesion size and the compositions were observed using Oil red O staining and immunofluorescent staining. Enzyme-linked immunosorbent assay (ELISA) was used to evaluate the levels of lipid profiles, oxidative stress, and inflammatory factors. Human aortic endothelial cells (HAoECs) were treated with oxLDL with or without TND for in vitro experiments.

**Results:**

TND treatment significantly suppressed the progression of atherosclerosis, as characterized with a smaller lesion size, less percentage of vascular smooth muscle cell proliferation, endothelial cell apoptosis, and macrophage infiltration. In addition, TND decreased the levels of lipid profiles, oxidative stress, and inflammatory factors in atherosclerosis. In vitro results showed that TND inhibited the apoptosis of endothelial cells via activating ERK and AKT pathway.

**Conclusions:**

Our study demonstrated that TND significantly protected from atherosclerosis via promoting endothelial cell survival and alleviating oxidative stress and inflammatory response, which may have become a treatment in atherosclerotic diseases.

## 1. Introduction

Carotid atherosclerosis, the main underlying cause of stroke, is one of the leading causes of mortality and morbidity worldwide [1, 2]. A lot of factors contributed to the pathogenesis of carotid atherosclerosis, including lipid deposition [3], macrophage infiltration [4], arterial smooth muscle cell proliferation [5], and endothelial cells (EC) apoptosis [6]. EC is a selectively permeable barrier between circulating blood and artery tissues and has been implicated as an early step in the pathogenesis of atherosclerosis [7]. Therefore, improving endothelial function is also critical for the prevention and treatment of carotid atherosclerosis.

Certain herbal extracts used in Traditional Chinese Medicine have shown significant potential for AS treatment [8, 9]. They have antioxidants and antiatherosclerotic effects [10]. Mechanistically, they modulated the activity of macrophages [11] and block the phenotype switch of VSMCs [11]. Tongnao decoction (TND), a Chinese decoction, had been designed and applied for the treatment of ischemic stroke since 2010 [12, 13]. Chuanxiong, one of the main bioactive ingredients, has various pharmacological properties, such as vascular-protective, antithrombotic, antiatherosclerosis, and anti-inflammatory effects (Chen et al., 2018). Previous studies showed that TND could enhance the angiogenic activity of endothelial cells [14] and upregulate some protective factors [15]. However, whether TND could inhibit carotid atherosclerosis progress is not clear.

The aim of this study was to evaluate the effects of TND in alleviating atherosclerosis progression and to elucidate its underlying mechanisms.

## 2. Materials and Methods

### 2.1. Preparation of Tongnao Decoction (TND)

TND is composed of Rhizoma arisaematis (lot# 20181225, Xintai Pharmaceutical Co. Ltd., Bozhou, China), Rhizoma Anemones altaicae (lot# 20181013, Xintai Pharmaceutical Co. Ltd., Bozhou, China), Rhizoma chuanxiong (lot# 20180922, Tongde Pharmaceutical Co. Ltd., Tongren, China), Rhizoma Gastrodiae (lot# 20181102, Tongde Pharmaceutical Co. Ltd., Tongren, China), Ramulus Uncariae Cum Uncis (lot# 20181218, Tongde Pharmaceutical Co. Ltd., Tongren, China), Bombyx batryticatus (lot# 20190122, Tongde Pharmaceutical Co. Ltd., Tongren, China), Hirudo (lot# 20190219, Jingquan Co. Ltd., Ma'anshan, China), and Radix et Rhizoma Rhodiolae Crenulatae (lot# 20181125, Xiehecheng Co. Ltd., Bozhou, China) (Supplementary Figure 1). The TND was prepared with the voucher specimen (2019–0318) deposited at the Department of Pharmacy in Jiangsu Provincial Hospital of Traditional Chinese Medicine. The methods and the composition were reported previously [14]. The UPLC analysis is shown in Supplementary Figure 2.

### 2.2. Animals

Male ApoE-/- mice (6-8 weeks old) were kept in a temperature-controlled room with a 12-hour light-dark cycle (Institute of Model Animals of Nanjing University, Nanjing). The mice were randomly divided into three groups: (1) sham group, (2) AS group, and (3) AS+TND group. The AS group underwent the operation that a silastic collar was placed around the right common carotid artery and fed on a Western diet containing 0.2% (wt/wt) cholesterol and 42% fat for 12 weeks. Briefly, a ventral milline incision (4-5 mm) was made in the neck. The right common carotid artery was exposed by blunt dissection. The collars were placed bilaterally around the common carotid arteries, and their axial edges were approximated by placement of 3 circumferential silk ties [16, 17]. The TND group was additionally given a gavage of Tongnao decoction (Nanjing University of Chinese Medicine) at a dose of 1 mg/kg/d. The sham group was fed on a chow diet. The protocol was approved by the Animal Care and Use Committee of Nanjing University of Chinese Medicine.

### 2.3. Atherosclerotic Lesion Analysis

After 12 weeks, mice were euthanized with cervical dislocation. The left and right common carotid artery were harvested under a stereomicroscope. They were immediately snap-frozen in liquid nitrogen after having been embedded in OCT compound (Tissue-Tek, Sakura Finetek). Transverse 4 *μ*m cryosections were prepared in a proximal direction from the carotid bifurcation and mounted in order on slides. Then, the slides were stained with Oil Red O solution (Sevicebio, Wuhan, China) and observed under a high-resolution camera. The percentage of total area stained by Oil Red O was determined as lesion area, and the area without nuclear staining within lesion area was determined as necrotic core. They were both semiquantified with ImageJ software (NIH, USA).

### 2.4. Immunofluorescent Staining

The deparaffinized slides were boiled in citrate buffer at 100°C for one hour for antigen retrieval and then were blocked in 1% FBS at room temperature followed by overnight incubation with primary antibodies. The primary antibodies, including CD31, *α*-SMA, and CD68 (CST, USA), were used to incubated with the sections, respectively, followed by incubated with PE-conjugated secondary antibody (Proteintech, Wuhan). Finally, cell nuclei were stained with DAPI. Images were captured and processed using a fluorescence microscope (IX 53, Olympus Corporation, Japan).

### 2.5. Enzyme-Linked Immunosorbent Assay (ELISA)

Mouse serum was collected by eyeball extirpating. The levels of lipid profiles (triglycerides (TG), total cholesterol (TC), high-density lipoprotein cholesterol (HDL-C), and low-density lipoprotein cholesterol (LDL-C)), redox-related proteins (superoxide dismutase (SOD), methylenedioxyamphetamine (MDA), and glutathione peroxidase (GPX)), and inflammatory factors (interleukin-1*β* (IL-1*β*), interleukin-6 (IL-6), tumor necrotic factor-*α* (TNF-*α*), vascular endothelial cell adhesion molecule-1 (VCAM-1), and intercellular adhesion molecule 1 (ICAM-1)) were measured using ELISA kits (Jin Yibai, Nanjing) according to the manufacturer's instructions. The optical densities of the samples were detected using a microplate reader (BIOTEK, USA) at a wavelength of 450 nm.

### 2.6. Cell Apoptosis

Human aortic endothelial cells (HAoECs) were obtained from Cell Applications (San Diego, USA) and cultured in endothelial cell growth medium (R&D systems, USA) supplemented with penicillin/streptomycin (Gibco, USA) at 37°C in 5% CO_2_. After reaching 70–80% confluence, they were cultured in starvation medium for 24 hours and then treated with oxLDL (30 *μ*g/mL) for an additional 24 hours in the presence or absence of TND (50 *μ*g/mL). Then, cells were resuspended in 200 *μ*L PBS and incubated with 5 *μ*L Annexin FITC and PI (Beyotime; China). The apoptotic cells were determined by flow cytometry (BD; USA).

### 2.7. Lactate Dehydrogenase Assay

When reaching a 60-70% converge, HAoECs were cultured in starvation medium for 24 hours and then treated with oxLDL (30 *μ*g/mL) and TND for an additional 24 hours. The concentrations of TND were set as 10, 20, 50, and 100 *μ*g/mL. Lactate dehydrogenase (LDH) release level was assessed by using a commercial kit (Keygen; China) according to the manufacturer's instructions. The experiments were performed in triplicates.

### 2.8. Western Blotting

Cells were lysed using RIPA buffer (Keygen; Jiangsu) and separated on SDS-PAGE gels (Millipore; USA). The membranes were incubated with primary rabbit anti-mouse antibodies including ERK, p-ERK, AKT, p-AKT, and GAPDH (Abcam, USA) at a dilution of 1 : 1000. After overnight incubation at 4°C, the membranes were subsequently incubated with HRP-conjugated rabbit anti-mouse IgG (1 : 10000) for 2 h at room temperature. The immunobands were visualized using an enhanced chemiluminescence detection kit (Beyotime; China).

### 2.9. Statistical Analysis

The data were presented as the mean ± SD. Statistical analysis was performed using ANOVA and Tukey's pos hoc test using Prism 8.0 (GraphPad; USA). *P* value < 0.05 was considered statistically significant.

## 3. Results

### 3.1. TND Mitigated the Progression of Atherosclerosis

**Figure 1:**
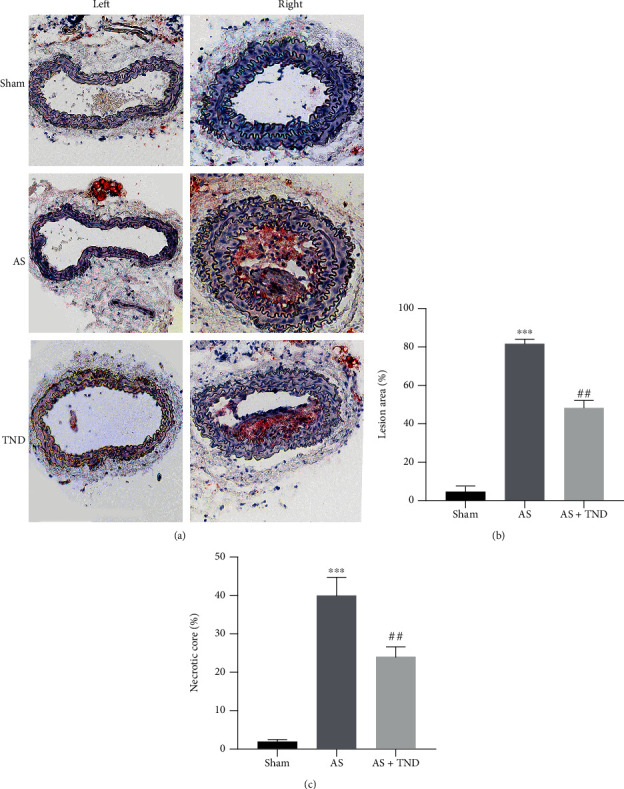
Tongnao decoction (TND) mitigated the progression of atherosclerosis in ApoE-deficient mice. The ApoE-/- mice were given a gavage of TND at a dose of 1 mg/kg per day (TND) or normal saline (AS) for 12 weeks after right carotid artery partial ligation. (a) Cross-sections of the left and right common carotid arteries were identified with the Oil Red O staining. The lesion area (b) and necrotic core size (c) of right carotid artery were quantified as the mean ± SEM based on Oil Red O staining. *N* = 6; ^∗∗∗^*P* < 0.001 vs. sham; ^##^*P* < 0.01 vs. AS.

To assess the therapeutic role of TND in atherosclerosis progression, ApoE-/- mice were given a gavage of TND (1 mg/kg per day) or normal saline (Supplementary Figure 3). The Oil Red O staining of the left and right common carotid arteries was performed between three groups, respectively. Compared with the AS group, the TND group had reduced atherosclerotic lesion area (Figure 1(a)). Quantitative results also showed that TND treatment decreased the lesion area size (Figure 1(b)) and necrotic core size (Figure 1(c)) of the right carotid artery.

### 3.2. TND Restored the Integrity of Intima and Inhibited Intima Hyperplasia within Carotid Artery

**Figure 2:**
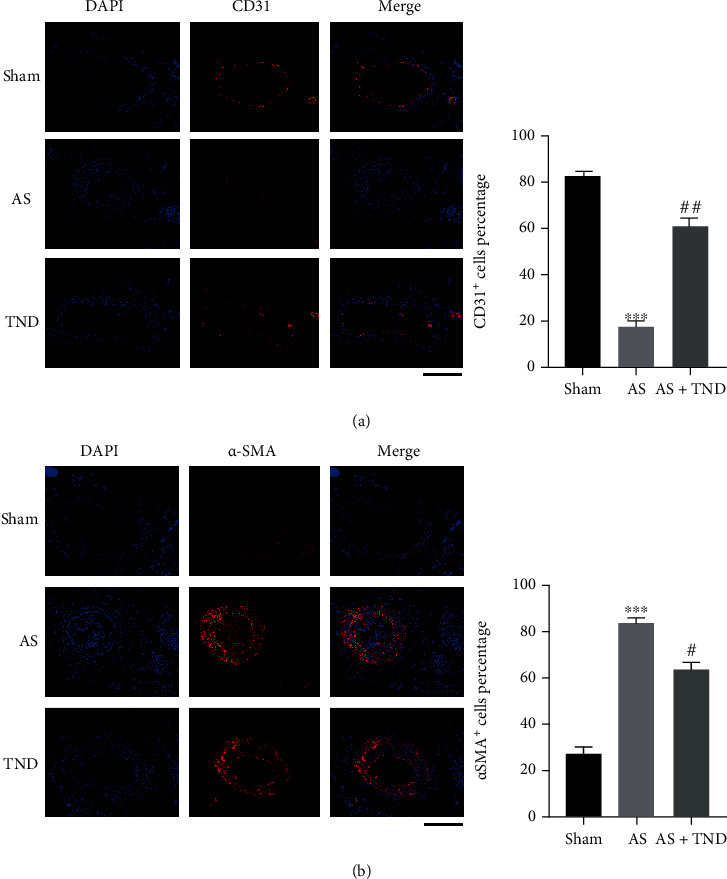
TND restored the apoptosis of endothelial cells and inhibited the proliferation of vascular smooth muscle cells within atherosclerotic artery. Endothelial cell proliferation after partial ligation of the carotid artery was assessed by immunofluorescence staining for CD31 (a), and vascular smooth muscle cell proliferation was assessed by immunofluorescence staining for *α*-SMA (b). The nuclei were stained blue with 4′-6-diamidino-2-phenylindole (DAPI). CD31 (stained in red) was used as an endothelial marker, and *α*-SMA (stained in red) was used as a smooth muscle marker. Scale bar indicates 100 *μ*m. ^∗∗∗^*P* < 0.001 vs. sham; ^##^*P* < 0.01 and ^#^*P* < 0.05 vs. AS.

Because endothelial cells and vascular smooth muscle cells are critical in the development of atherosclerosis, we investigated the effect of TND on endothelial cells and vascular smooth muscle cells. Immunofluorescence staining revealed that there were less CD31^+^ endothelial cells (Figure 2(a)) and more *α*-SMA^+^ smooth muscle cells (Figure 2(b)) in the AS group, while TND restored the number of endothelial cells and smooth muscle cells, suggesting that TND could promote endothelial cell survival while inhibit vascular smooth muscle cell proliferation.

### 3.3. TND Alleviated Macrophage Infiltration within Atherosclerotic Carotid Artery

**Figure 3:**
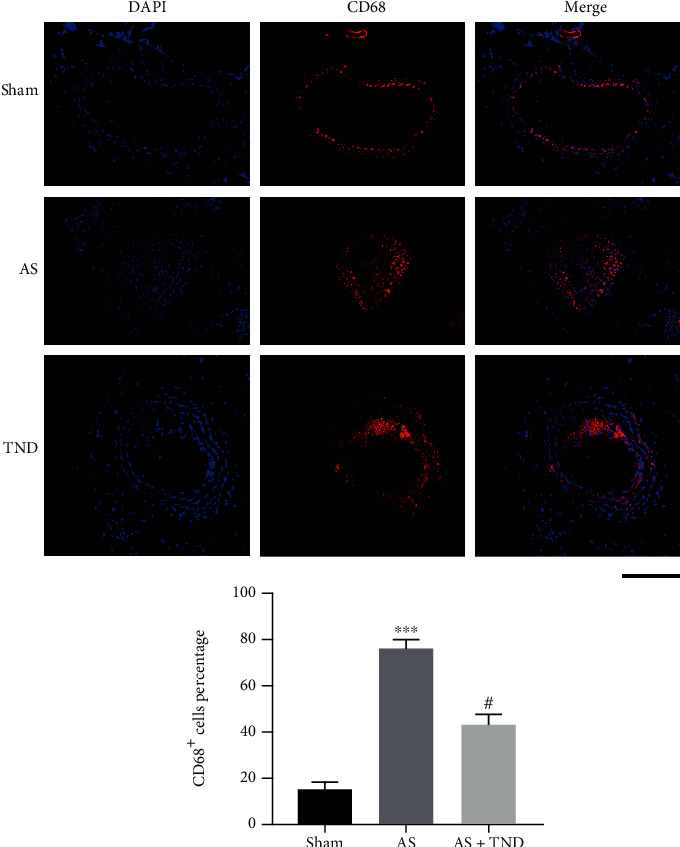
TND alleviated macrophage infiltration within atherosclerotic carotid artery. The macrophage infiltration was assessed by immunofluorescence staining for CD68. The nuclei were stained blue with 4′-6-diamidino-2-phenylindole (DAPI). CD68 (stained in red) was used as a macrophage marker. Scale bar indicates 100 *μ*m. ^∗∗∗^*P* < 0.001 vs. sham; #*P* < 0.01 vs. AS.

Macrophage infiltration was a trigger of unstable carotid atherosclerosis. Immunofluorescent imaging results showed that CD68^+^ cells were reduced in TND group compared with the AS group (Figure 3), revealing that TND alleviated macrophage infiltration in atherosclerotic carotid artery.

### 3.4. TND Modulated the Lipid Profiles in Atherosclerosis

**Figure 4:**
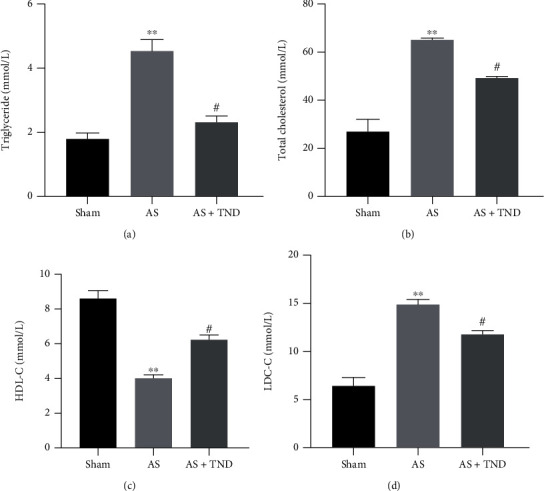
TND modulated the lipid profiles of circulating blood. The serum levels of triglycerides (a), total cholesterol (b), HDL-C (c), and LDL-C (d) were measured using enzyme linked immunosorbent assay. One-way analysis of variance with the Bonferroni post hoc test was used. *N* = 6; ^∗∗^*P* < 0.01 vs. sham; #*P* < 0.05 vs. AS.

Excess lipid deposits contributed to the initiation of atherosclerosis. Therefore, we evaluated the effect of TND on lipid profiles of circulating blood. We used ELISA to detect the levels of serum triglycerides (Figure 4(a)), total cholesterol (Figure 4(b)), high-density lipoprotein (Figure 4(c)), and low-density lipoprotein (Figure 4(d)). Our results showed that TND treatment decreased the levels of triglycerides, total cholesterol, and low-density lipoprotein, while it increased the levels of high-density lipoprotein.

### 3.5. TND Alleviated the Level of Inflammation Response and Oxidative Stress

**Figure 5:**
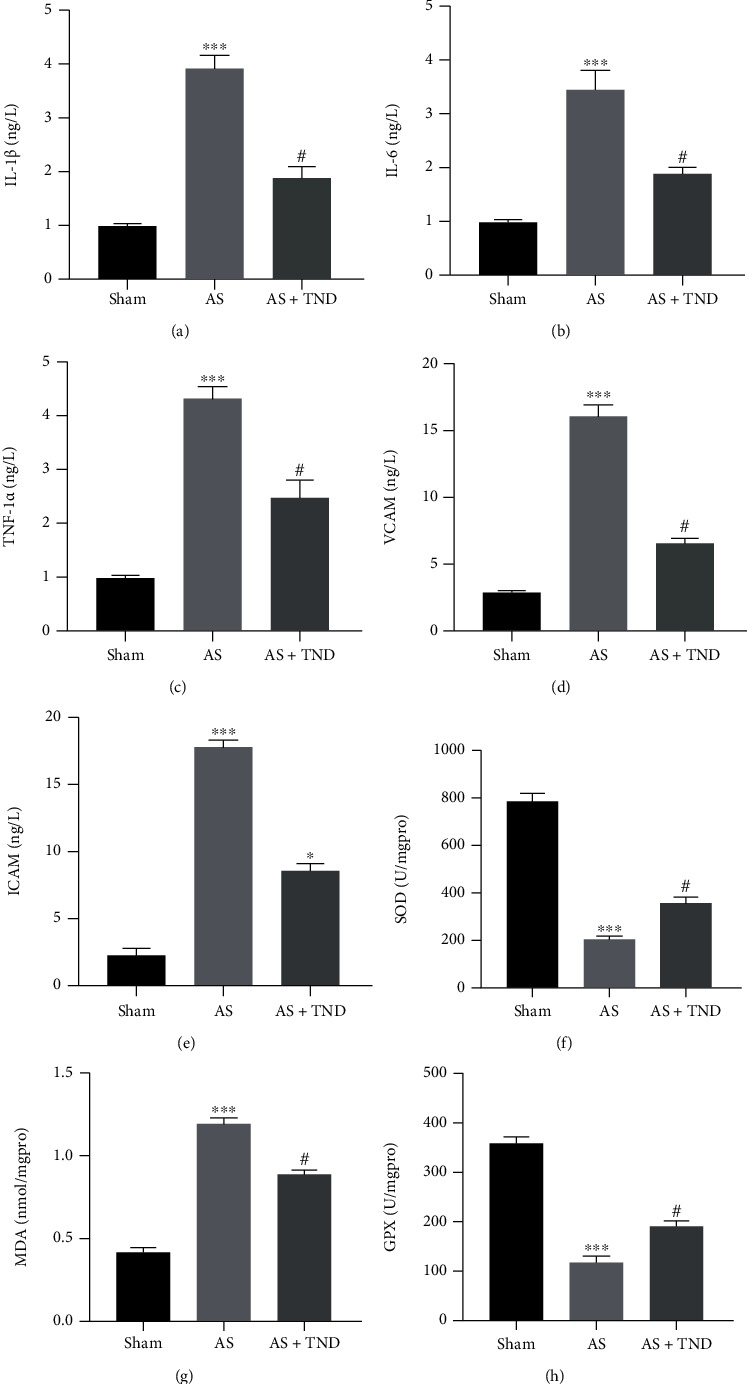
TND alleviated the systemic level of inflammation response and oxidative stress. The serum levels of IL-1*β* (a), IL-6 (b), TNF-*α* (c), VCAM (d), and ICAM (e) as well as the activity of SOD (f), MDA (g), and GPX (h) were measured using enzyme linked immunosorbent assay. One-way analysis of variance with the Bonferroni post hoc test was used. *N* = 6; ^∗∗∗^*P* < 0.001 vs. sham; #*P* < 0.05 vs. AS.

Chronic inflammation and oxidative stress contributed to the development of atherosclerosis. We used ELASA to assess the levels of inflammatory factors (IL-1*β*, IL-6, TNF-*α*, VCAM, and ICAM) (Figures 5(a)–5(e)) and oxidative stress-related markers (SOD, MDA, and GPX) (Figures 5(f)–5(h)). These results suggested that TND could alleviate the systemic level of inflammation response and oxidative stress.

### 3.6. TND Inhibited the Apoptosis of HAoECs via Activating ERK and AKT Pathway

**Figure 6:**
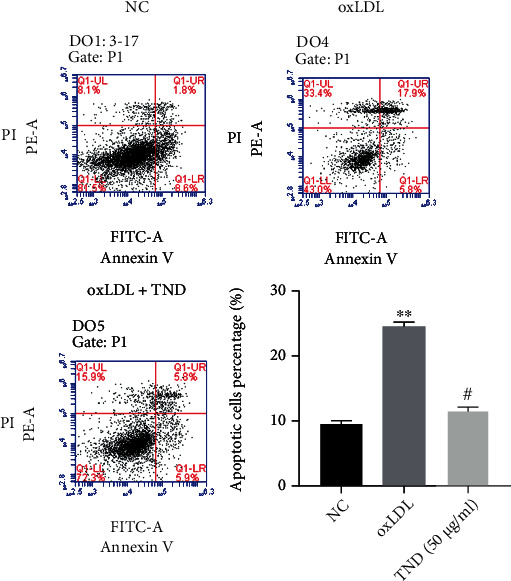
TND inhibited the apoptosis of human aortic endothelial cells (HAoECs). HAoECs were treated with oxLDL (30 *μ*g/mL) and with or without TND (50 *μ*g/mL) for an additional 24 hours. The effect of TND on HAoECs apoptosis was measured using Annexin V/PI flow cytometry. Apoptosis was assessed by the ratio of Annexin-positive cells to total cells. The data were expressed as the mean ± SEM of three independent experiments. ^∗∗^*P* < 0.01 vs. sham; #*P* < 0.05 vs. AS.

**Figure 7:**
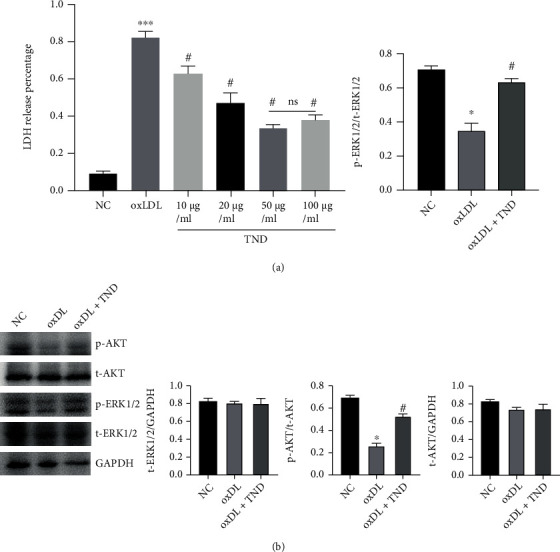
TND promoted the survival of HAoECs via activating ERK and AKT pathway. HAoECs were treated with oxLDL (30 *μ*g/mL) and with or without TND for an additional 24 hours. (a) The concentrations of TND were set as 10, 20, 50, and 100 *μ*g/mL. The cytoprotective effects of TND were measured using LDH release kits. (b) The concentrations of TND were set as 50 *μ*g/mL. The prosurvival pathways, ERK and AKT signaling, were determined by immunoblotting. ^∗∗∗^*P* < 0.001 vs. sham; #*P* < 0.05 and ##*P* < 0.01 vs. oxLDL.

As we all know, endothelial cell apoptosis contributed to the progression of atherosclerosis. As shown in Figure 6, TND significantly decreased the percentage of Annexin V^+^/PI^−^ and Annexin V^+^/PI^+^-positive cells, suggesting an antiapoptotic role of TND. As shown in Figure 7(a), TND decreased the LDH release in oxLDL-treated HAoECs in a dose-dependent manner, suggesting the cytoprotective effect of TND. To investigate the downstream pathway, we performed immunoblotting analysis of TND-treated cells. The ratios of p-ERK/t-ERK and p-AKT/t-AKT were decreased in the DOX group while TND restored their expression level (Figure 7(b)). These results suggested that TND could promote endothelial cells survival.

## 4. Discussion

In this study, we showed for the first time that TND could alleviate the progression of carotid atherosclerosis. Specially, TND inhibited systemic and local inflammation response, oxidative stress, and endothelial apoptosis in atherosclerotic carotid artery. In vitro experiments showed that TND promoted the survival of aortic endothelial cells via activating AKT and ERK signaling pathway.

Atherosclerosis is characterized by a lipoprotein-driven inflammatory disorder and oxidative stress leading to a plaque formation at specific sites of the artery [18, 19]. Oxidative stress, which is exemplified by the overproduction of reactive oxygen species (ROS) and oxidized low-density lipoprotein (ox-LDL), has a pivotal role in the progression of atherosclerosis. The anti-oxidant and anti-inflammatory actions of nutraceuticeuticals and plant ingredients play a significant role in capturing free radicals and reducing endothelial risk factors associated with AS [20]. In according to previous publications, we also confirmed that TND could play a beneficial role via increasing the antioxidant enzymes. In addition, macrophage activation was an important pathophysiological process in atherosclerosis [21]. In our study, we observed that TND alleviated macrophage infiltration, restricting the formation of atherosclerotic plaques [22]. Most importantly, endothelial injury was the initial step in the development of atherosclerosis [23]. TND has the potential to inhibit apoptosis of endothelial cells and promote the survival of endothelial cells via activating ERK and AKT pathway, which was consistent with the previous results [14].

In conclusion, our study showed that TND could protect carotid atherosclerosis progression by promoting the survival of endothelial cells and inhibiting macrophage infiltration, which provided a direction for the clinical application of TND in carotid atherosclerosis.

## Data Availability

All data are available from the corresponding author upon reasonable request.

## Abbreviations

ECM: Extracellular matrix
ELISA: Enzyme-linked immunosorbent assay
GPX: Glutathione peroxidase
HAoECs: Human aortic endothelial cells
HDL-C: High-density lipoprotein cholesterol
ICAM-1: Intercellular adhesion molecule 1
IL-1*β*: Interleukin-1*β*
IL-6: Interleukin-6
LDL-C: Low-density lipoprotein cholesterol
MDA: Methylenedioxyamphetamine
ox-LDL: Oxidized low-density lipoprotein
ROS: Reactive oxygen species
SOD: Superoxide dismutase
TC: Total cholesterol
TG: Triglycerides
TND: Tongnao decoction
TNF-*α*: Tumor necrotic factor-*α*
VCAM-1: Vascular endothelial cell adhesion molecule-1.

## Supplementary Materials

Supplementary Materials Supplementary Figure 1: the compositions of Tongnao decoction (TND) and percentage in a prescription. Supplementary Figure 2: the chromatographic fingerprint of TND. A typical chromatogram contains 83 peaks representing chemical markers of different component Chinese medicines in TND. A. positive ion mode, B. negative ion mode. Supplementary Figure 3: the flow chart of experimental design.
