# Supplementary material for: Tongnao Decoction (TND) Alleviated Atherosclerosis by Playing Lowering Lipid, Anti-Inflammatory, and Antioxidant Roles
Source: Oxid Med Cell Longev. 2022 May 25;2022:6061197. doi: 10.1155/2022/6061197 (PMC9159833; doi:10.1155/2022/6061197)
Supplement: Supplementary Materials — Supplementary Figure 1: the compositions of Tongnao decoction (TND) and percentage in a prescription. Supplementary Figure 2: the chromatographic fingerprint of TND. A typical chromatogram contains 83 peaks representing chemical markers of different component Chinese medicines in TND. A. positive ion mode, B. negative ion mode. Supplementary Figure 3: the flow chart of experimental design. [file 6061197.f1.pdf]

Compositions	Percentage (%)
Ramulus Uncariae Cum Uncis	30
Radix et Rhizoma Rhodiolae Crenulatae	15
Rhizoma chuanxiong	10
Rhizoma arisaematis	10
Rhizoma Anemones altaicae	10
Rhizoma Gastrodiae	10
Bombyx batryticatus	10
Hirudo	5

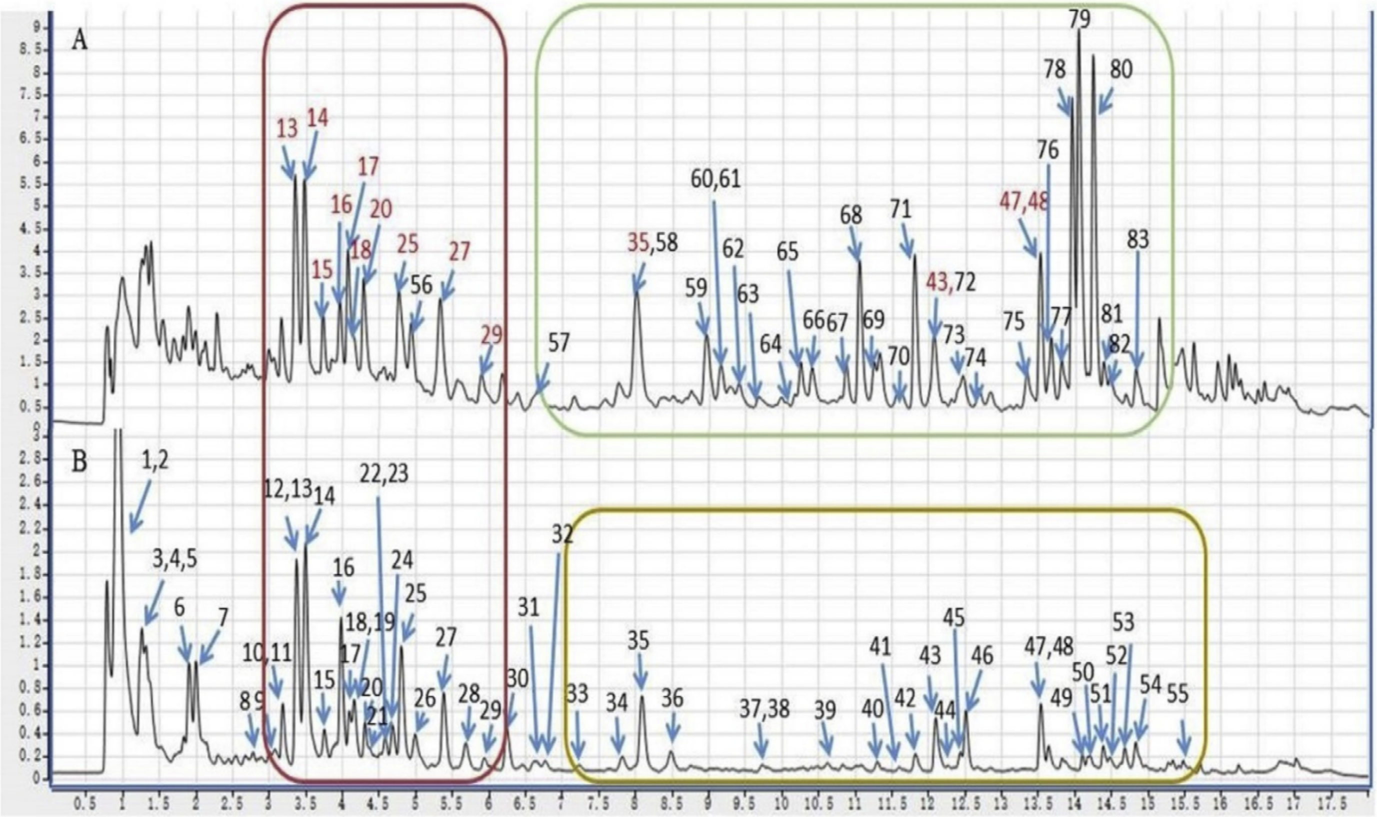

1.Sucrose
2.Quinic acid
3.Vanillic acid
4.Citric acid
5.2-Hydroxy-4-methylvaleric acid
6.Gastrodin
7.Gallic acid
8.Parishin E
9.Dihydroxybenzoic acid
10.NeoChlorogenic acid
11.Nonioside A
12.Methyl-galloylgalactarate
13.Salidroside
14.Crenuation
15.S-(4-hydroxybenzyl)-glutathione
16.Chlorogenic acid
17.Creoside I
18.Crypto chlorogenic acid
19.Catechin
20.Creoside I
21.Tri-O-galloyl-glucose
22.Creoside II
23.Vanillic acid
24.Caffeic acid
25.Parishin B
26.Catechin
27.Parishin C
28.galloyl-salidroside
29.Paeoniflorin
30.unknow
31.Coumaric acid
32.Glucopyranosyl-hydroxyvincoside lactam glucoside/isomer
33.Tetra-O-galloyl-glucose
34.Rutin
35.Parishin A
36.Epigallocatechin gallate
37.unknown
38.Penta-galloylglucose
39.unknow
40.Rhodiosin
41.unknow
42.Epicatechin
43.Rhodiosin/isomer
44.Nepitrin
45.Salvianolic acid B
46.Quercitrin
47.Rhodiooctanoside
48.Rhodiolatuntoside
49.unknow
50.unknow
51.unknow
52.unknow
53.unknow
54.unknow
55.Methoxy-tetrahydronaphthalenecarboxylic acid
56.Dihydro-O-demethyl-O-β-D-glucopyranosyl hirsutine
57.Glucopyranosyl-hudroxyvincoside lactam glucoside
58.Cadambine
59.3α-Dihydrocadambine
60.Isorhynchophyllinic acid
61.3α-Dihydrocadambine/isomer
62.Dihydro-demethylhirsutine
63.23-O-Demethylhirsutine
64.Rhynchophyllinic acid
65.Isocorynoxeine
66.Demethyl-glucopyranosyl-isocorynoxeine
67.Rhynchophylline
68.Corynoxeine
69.Demethylhirsutine/isomer
70.Dihydro-demethylhirsutine/isomer
71.Isorhynchophylline
72.Demethylhirsuteine/isomer
73.Ajmalicine/isomer
74.Ajmalicine/isomer
75.Ajmalicine/isomer
76.Demethylhirsutine/isomer
77.Hirsuteine
78.Geissoschizine methyl
79.Corynantheine
80.Hirsutine
81.Strictosamide
82.Vallesiachotamine/isomer
83.Ajmalicine/isomer

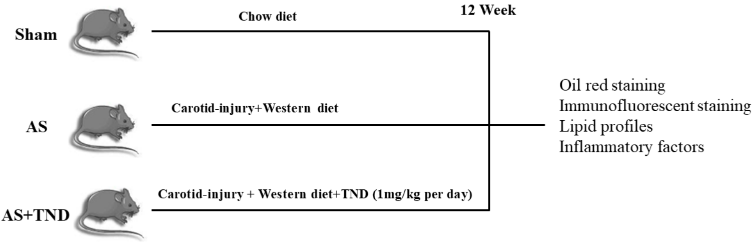
Sham
Chow diet
12 Week
AS
Carotid-injury+Western diet
AS+TND
Carotid-injury + Western diet+TND (1mg/kg per day)
Oil red staining
Immunofluorescent staining
Lipid profiles
Inflammatory factors
